# Impact of genetic mutations on prognosis and chemotherapy efficacy in advanced appendiceal carcinoma: insights from the nationwide Japanese comprehensive genomic profiling test database

**DOI:** 10.1007/s10147-025-02724-2

**Published:** 2025-02-28

**Authors:** Sakura Hiraide Taniguchi, Masanobu Takahashi, Shih-Wei Chiu, Keigo Komine, Shonosuke Wakayama, Ryunosuke Numakura, Yuya Yoshida, Yuki Kasahara, Kota Ouchi, Hiroo Imai, Ken Saijo, Hidekazu Shirota, Chikashi Ishioka

**Affiliations:** 1 Department of Medical Oncology, Tohoku University Hospital, Sendai, Japan; 2 Department of Clinical Oncology, Tohoku University Graduate School of Medicine, 4-1, Seiryo-Machi, Aoba-Ku, Sendai, Miyagi 980-8575 Japan; 3 Clinical Research Data Center, Tohoku University Hospital, Sendai, Japan

**Keywords:** Appendiceal carcinoma, Comprehensive genomic profiling test, Systemic chemotherapy, Genome, Prognosis

## Abstract

**Background:**

Appendiceal carcinoma (AC) is a rare malignancy and has distinct genomic features, but their impact on prognosis and chemotherapy efficacy requires further investigation.

**Methods:**

This retrospective study analyzed patients with advanced AC from the Japanese nationwide comprehensive genomic profiling test database, the Center for Cancer Genomics and Advanced Therapeutics (C-CAT) database, focusing on genetic alterations and their associations with clinical outcomes.

**Results:**

Of the 314 patients, the histological types Queryincluded adenocarcinoma (Ad) (51.9%), mucinous adenocarcinoma (MAd) (30.3%), goblet cell adenocarcinoma (12.4%), and signet-ring cell adenocarcinoma (5.4%). The most common mutations were *KRAS* (52.5%), *TP53* (49.4%), *SMAD4* (18.8%), and *GNAS* (17.2%). *KRAS* mutations were most frequent in MAd (68.4%) and Ad (58.9%), whereas *TP53* mutations were mostly prevalent in Ad (62.6%). We classified patients into molecular subtypes based on the presence of mutations and analyzed differences in overall survival (OS) by molecular subtype. Patients with *TP53*-mutant (mut) dominant tumors (all *TP53*-mut) and *KRAS*-mut focused tumors (*TP53*-wild-type (wt)/*GNAS*-wt/*KRAS*-mut/any *SMAD4*) showed a poorer median OS compared with those with *GNAS*-mut focused tumors (*TP53*-wt/*GNAS*-mut/any *KRAS* /any *SMAD4*) (median 47.4 and 37.5 months vs. not reached; *p* = 0.01 and *p* = 0.01, respectively). *TP53* mutation was associated with poor time to treatment failure and OS with the oxaliplatin-based regimen for first-line chemotherapy.

**Conclusions:**

This study suggested that the genetic mutations influenced the prognosis and chemotherapy efficacy in AC.

**Supplementary Information:**

The online version contains supplementary material available at 10.1007/s10147-025-02724-2.

## Introduction

Appendiceal carcinoma (AC) is a rare malignancy and presents with a variety of histologic findings and clinical presentations [1, 2]. This cancer comprises multiple histopathologic subtypes. The 5th edition 2019 WHO Classification of Tumors categorizes epithelial tumors of the appendix into serrated lesions and polyps, mucinous neoplasms, adenocarcinoma, undifferentiated carcinoma, goblet cell adenocarcinoma (GCA), and neuroendocrine neoplasms. Additionally, adenocarcinoma is divided into mucinous adenocarcinoma (MAd) and signet-ring cell adenocarcinoma (SRC) [3, 4]. In contrast, the OncoTree classification categorizes appendiceal tumors into appendiceal adenocarcinoma, low-grade appendiceal mucinous neoplasm, and well-differentiated neuroendocrine tumor of the appendix. Furthermore, appendiceal adenocarcinoma is classified as colonic type adenocarcinoma of the appendix, mucinous adenocarcinoma of the appendix, signet-ring cell type of the appendix, and goblet cell adenocarcinoma of the appendix [5].

No standard chemotherapy guidelines specifically for advanced AC in the absence of a randomized phase III study have been established [4]. The chemotherapy regimens for colorectal cancer (CRC) are often applied to patients with inoperable advanced AC, as recommended by the National Comprehensive Cancer Network (NCCN) guidelines [6, 7].

Recent molecular profiling studies have confirmed that AC has unique genomic features that differ from those of CRC [8–12]. Some studies have also reported associations between genomic alterations and clinicopathological features [8, 9, 11, 12], but few studies include data on chemotherapy.

AC is rare and has diverse histological patterns, leading to varying diagnoses and opinions among different pathologists [2, 13]. Therefore, there is a need for an objective subtype classification that can accurately predict the prognosis of AC and stratify patients. The question arises as to whether subtyping by genomic alterations is useful in predicting the prognosis and chemotherapy efficacy.

In this study, using the Center for Cancer Genomics and Advanced Therapeutics” (C-CAT) database in Japan [14, 15], we aimed to clarify the association between the genomic features and prognosis of AC and the efficacy of its treatment and to determine whether subtyping based on genomic features is useful as a prognostic biomarker.

## Patients and methods

### Patients

**Fig. 1:**
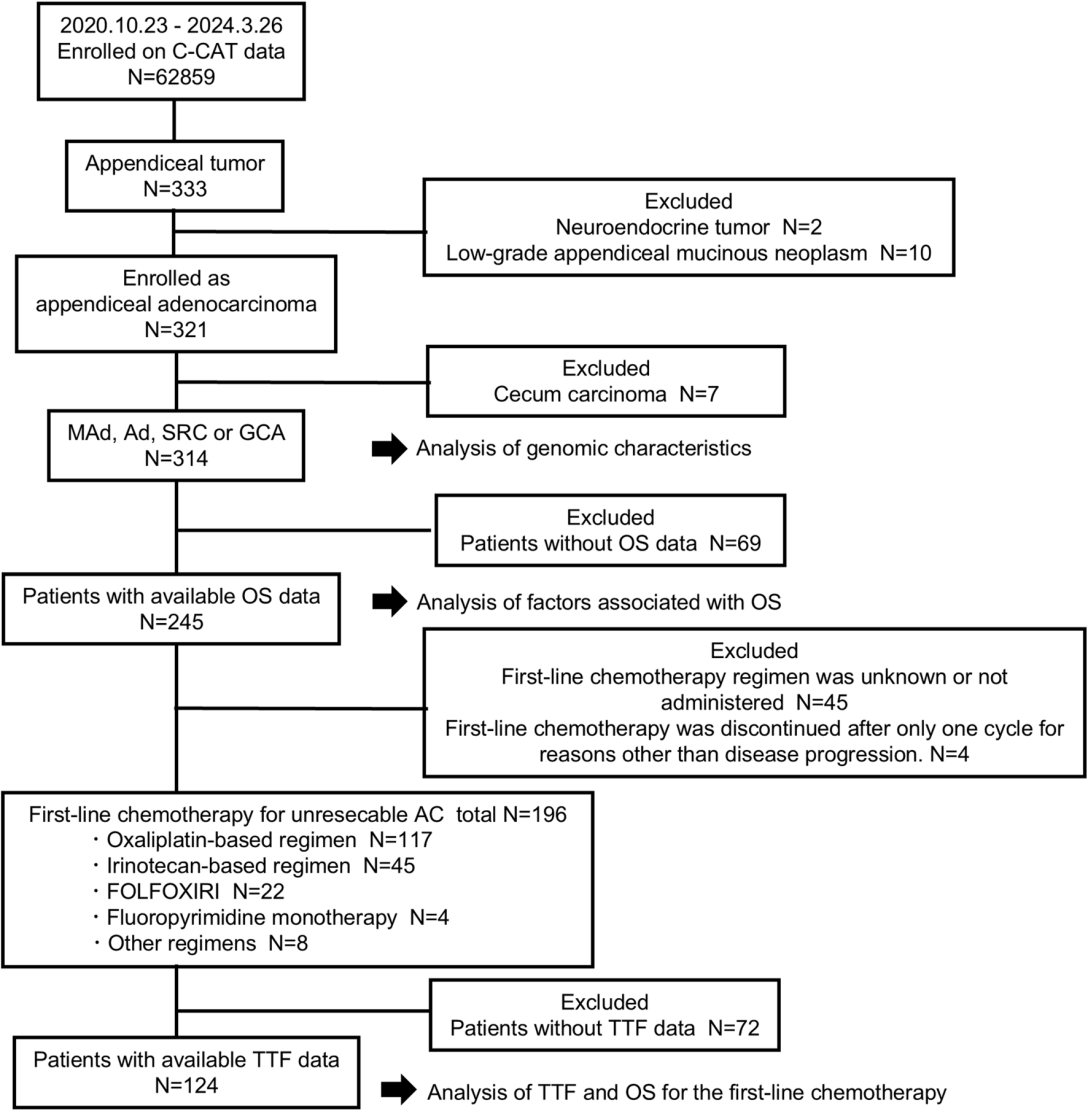
CONSORT diagram of the study. The diagram shows the flow of patients included in the study and patients excluded from the analyses. *C-CAT* the center for cancer genomics and advanced therapeutics*, Ad* adenocarcinoma, *MAd* mucinous adenocarcinoma, *SRC* signet-ring cell adenocarcinoma, *GCA* goblet cell adenocarcinoma, *OS* overall survival, *TTF* time to treatment failure, *AC* appendiceal cancer

The present retrospective observational study was conducted using the data of 321 AC patients who were registered in the C-CAT database between 23 October 2020 and 26 March 2024. The participants underwent gene-panel testing, FoundationOne CDx (F1 CDx; Foundation Medicine Inc., Cambridge, USA), OncoGuide NCC Oncopanel System (NCC Oncopanel) (Sysmex Co., Ltd., Kobe, Japan), FoundationOne Liquid CDx (F1 Liquid), Guardant360 (Guardant Health, Redwood City, USA), or GenMine TOP (Konica Minolta Inc., Tokyo, Japan). Based on the cancer classification of the OncoTree platform [5], 333 patients were enrolled as appendiceal tumors. We excluded two patients with well-differentiated neuroendocrine tumor of the appendix and ten patients with low-grade mucinous tumors in the following analysis, because of the small number of patients (Fig. 1). A total of 321 cases were registered as “appendiceal adenocarcinoma,” “mucinous adenocarcinoma of the appendix,” “colonic type adenocarcinoma of the appendix,” “signet-ring cell type of the appendix,” “goblet cell carcinoid of the appendix” in the 20,200,401 OncoTree version, and “goblet cell adenocarcinoma of the appendix” in the 20,230,725 OncoTree version. Since the OncoTree codes of certain cases were appendiceal adenocarcinoma, some of which do not reflect histological information, we reviewed the data of the pathological diagnosis name and OncoTree codes, and reclassified cases into adenocarcinoma (Ad), MAd, SRC, and GCA. We found that 314 patients had a diagnosis of AC after seven patients with cecum carcinoma were excluded.

### Methods

This study obtained the clinicopathological data from the study participants. Time to treatment failure (TTF) was defined as the time from the start of chemotherapy to its discontinuation due to any cause. In the analysis of patients, which included those who underwent chemotherapy and those who did not, overall survival (OS) was defined as the time from the initial diagnosis to death or last follow-up. In the analysis of chemotherapy efficacy, OS was defined as the time from the start of chemotherapy to death or last follow-up. Only the variants reported as “oncogenic,” “pathogenic,” “likely oncogenic,” and “likely pathogenic” in the clinical annotation of the C-CAT findings [13] were extracted, while variants of unknown significance were excluded. The gene mutational landscape was visualized using the OncoPrinter platform on cbioportal.org [16].

### Statistical analysis

The statistical analyses were performed using JMP^®^ pro 15 (SAS Institute Inc., Cary, NC, USA). The categorical variables were compared using Fisher’s exact test. A comparative survival analysis and median calculation were performed using the Kaplan–Meier method, whereas the significance between the two groups was verified using the log-rank test. Moreover, univariable and multivariable analyses were conducted using the Cox proportional hazards model. Covariates with Wald test *p* values ≤ 0.10 in the univariable analysis, as well as those clinically inferred to be potentially prognostically relevant, were included in the multivariable analysis. Statistical significance was set at a *p* value of < 0.05.

### Ethical statement.

This study was approved by the Ethics Committee of Tohoku University Hospital (No. 2024-1-161) and the review board of the C-CAT (No. CDU2022-016E03).

## Results

### Genomic characteristics

A total of 314 patients’ data were analyzed to characterize the genome of AC. All patients underwent a comprehensive genomic profiling (CGP) test under the National Health Insurance System [15] and were diagnosed with metastatic or locally advanced AC that was considered eligible for chemotherapy.

Table 1 shows the clinical characteristics of the patients. The common histological type was Ad (51.9%), followed by MAd (30.3%), GCA (12.4%), and SRC (5.4%).

**Table 1:** Clinicopathologic characteristics of the patients

Characteristics	All patients (*n* = 314)	%	Patients with available OS data (*n* = 245)	%
	*n*		*n*	
Gender				
Male	143	45.5	115	46.9
Female	171	54.5	130	53.1
Median age, years (range)	58 (23–85)		57 (23–84)	
ECOG-PS^a^				
0	172	54.8	133	54.3
1	118	37.6	99	40.4
2	6	1.9	6	2.4
Unknown	18	5.7	7	2.9
Histology				
Ad	163	51.9	117	47.8
MAd	95	30.3	83	33.9
SRC	17	5.4	14	5.7
GCA	39	12.4	31	12.7
CGP test				
FoundationOne CDx	239	76.1	183	74.7
FoundationOne Liquid	41	13.1	35	14.3
NCC Oncopanel	30	9.6	26	10.6
Guardant360	2	0.6	1	0.4
GenMine TOP	2	0.6	0	0
First-line chemotherapy				
Oxaliplatin-based regimen	131	41.7	117	47.8
Irinotecan-based regimen	49	15.6	45	18.4
FOLFOXIRI	26	8.3	22	9.0
Fluoropyrimidine monotherapy	6	1.9	4	1.6
Other regimens	8	2.5	8	3.3
Others^b^	94	29.9	49	20.0
Previous chemotherapy lines^c^				
0	17	5.4	15	6.1
1	96	30.6	87	35.5
2	66	21.0	59	24.1
3 or more	62	19.7	56	22.9
Unknown	73	23.2	28	11.4

*OS*, overall survival*, ECOG-PS* Eastern Cooperative Oncology Group Performance Status*, CGP* comprehensive genomic profiling, *Ad* adenocarcinoma, *MAd* mucinous adenocarcinoma, *SRC* signet-ring cell adenocarcinoma, *GCA* goblet cell adenocarcinoma

^a^PS at the time of registration in the Center for Cancer Genomics and Advanced Therapeutics database

^b^This group includes 45 patients whose chemotherapy regimen was unknown or not administered, and 4 patients whose chemotherapy was discontinued after only one cycle for reasons other than disease progression

^c^Number of lines of prior chemotherapy for unresectable settings received before the CGP test was performed. Adjuvant chemotherapy is excluded

**Fig. 2:**
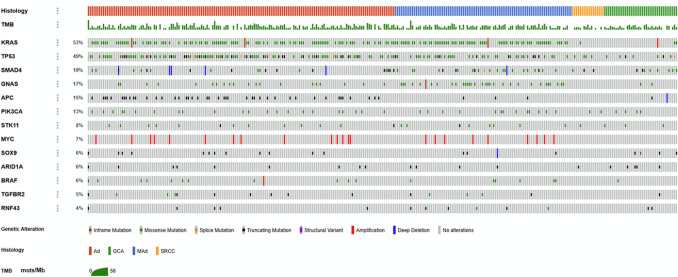
Genomic landscape of AC. OncoPrint shows the common genetic alterations, histology, and TMB (muts/Mb). *TMB* tumor mutation burden, *Ad* adenocarcinoma, *MAd* mucinous adenocarcinoma, *SRC* signet-ring cell adenocarcinoma, *GCA* goblet cell adenocarcinoma

**Fig. 3:**
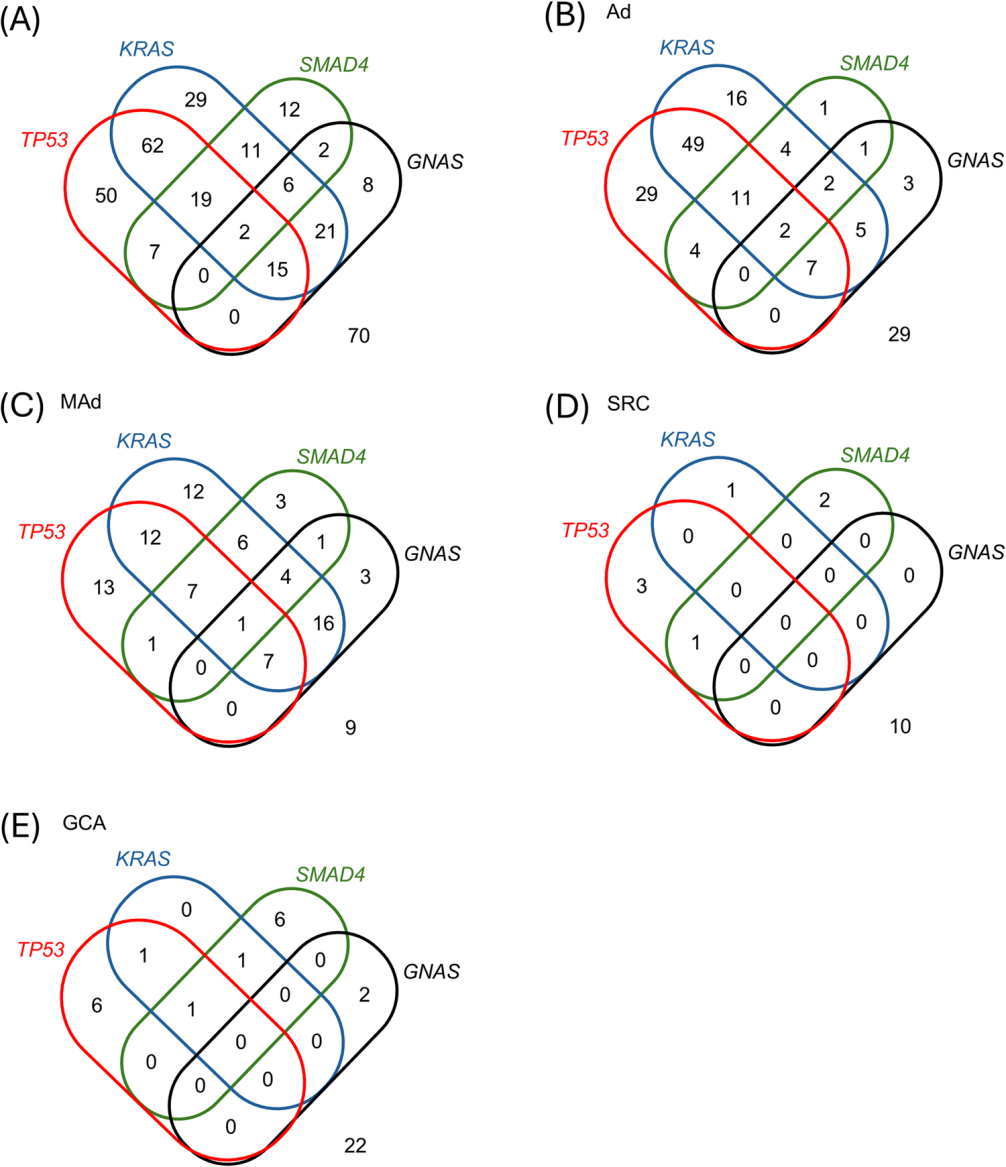
Venn diagrams showing the overlap of genetic mutation profiles. Venn diagrams illustrating the overlap of the KRAS, GNAS, TP53, and SMAD4 gene mutation profiles across the entire cohort (**a**), adenocarcinoma (**b**), mucinous adenocarcinoma (**c**), signet-ring cell adenocarcinoma (**d**), and goblet cell adenocarcinoma (**e**)

Genomic alterations were identified in 293/314 (93.3%) patients. Of the 293 patients, 157 distinct genes were altered (Table S1). The most frequently mutated genes were *KRAS* (52.5%), *TP53* (49.4%), *SMAD4* (18.8%), *GNAS* (17.2%), *APC* (15.3%), and *PIK3CA* (13.1%) (Fig. 2). The Venn diagrams illustrating the overlap of genetic mutation profiles for *KRAS*, *TP53*, *SMAD4,* and *GNAS* across the entire cohort and each histological subtype are shown in Fig. 3.

The prevalence of *KRAS*, *TP53*, and *GNAS* mutations varied by histology (*p* < 0.0001 for all three mutations, Table 2). The *KRAS* mutations were mostly common in MAd (68.4%) and Ad (58.9%), indicating lower frequencies in SRC (5.9%) and GCA (7.7%). The percentage difference between MAd and SRC was 62.5% (95% confidence interval (CI) 40.9–74.1), between MAd and GCA was 60.7% (95% CI 45.3–71.3), between Ad and SRC was 53.0% (95% CI 32.6–64.0), and between Ad and GCA was 51.2% (95% CI 37.2–60.8). The *TP53* mutations were mostly prevalent in Ad (62.6%), followed by MAd (43.2%), indicating lower rates in SRC (23.5%) and GCA (20.5%). The percentage difference between Ad and MAd was 19.4% (95% CI 6.8–31.4), between Ad and SRC was 39.1% (95% CI 15.0–57.2), and between Ad and GCA was 42.1% (95% CI 25.8–55.1). The *GNAS* mutations were more frequent in MAd (33.7%) than in Ad (12.3%), SRC (0%), and GCA (5.1%). The percentage difference between MAd and Ad was 21.4% (95% CI 10.6–32.0), between MAd and SRC 33.7% (95% CI 15.0–42.5), and between MAd and GCA 28.6% (95% CI 14.4–39.1). In contrast, the frequency of the *SMAD4* and *PIK3CA* mutations did not differ significantly among the histological types.

**Table 2:** Prevalence of gene mutations in each histology

Gene	MAd (*n* = 95)		Ad (*n* = 163)		SRC (*n* = 17)		GCA (*n* = 39)	
	*n*	%	*n*	%	*n*	%	*n*	%
*KRAS*	65	68.4	96	58.9	1	5.9	3	7.7
*TP53*	41	43.2	102	62.6	4	23.5	8	20.5
*SMAD4*	23	24.2	25	15.3	3	17.6	8	20.5
*GNAS*	32	33.7	20	12.3	0	0	2	5.1
*APC*	3	3.2	43	26.4	0	0	2	5.1
*PIK3CA*	13	13.7	22	13.5	2	11.8	4	10.3
*STK11*	8	8.4	13	8.0	1	5.9	3	7.7
*MYC*	9	9.5	14	8.6	0	0	0	0
*SOX9*	4	4.2	12	7.4	0	0	4	10.3
*ARID1A*	4	4.2	9	5.5	1	5.9	5	12.8
*BRAF*	6	6.3	12	7.4	0	0	1	2.6
*TGFBR2*	5	5.3	10	6.1	0	0	1	2.6

*Ad* adenocarcinoma, *MAd* mucinous adenocarcinoma, *SRC* signet-ring cell adenocarcinoma, *GCA* goblet cell adenocarcinoma

Four patients (1.3%) were diagnosed with microsatellite-instable tumors. Furthermore, a total of 22 patients (7.0%) showed tumor mutation burden-high (TMB-high, > 10 mutations/Mb) tumors as confirmed by the tissue-based CGP test.

### Survival prediction using genetic mutation patterns

The factors associated with the survival of the AC patients were assessed. Of the 314 AC patients registered in the C-CAT database, 245 with available OS data were used for the survival analysis.

Tables 1 and S1 show the clinicopathologic and genomic characteristics of the 245 patients.

A total of 196 patients’ data were analyzed for the first-line chemotherapy for unresectable AC, excluding 45 patients whose chemotherapy regimen was unknown or not administered and 4 patients whose chemotherapy was discontinued after only one cycle for certain reasons other than disease progression. The first-line chemotherapy regimens included the oxaliplatin-based regimen (*n* = 117), irinotecan-based regimen (*n* = 45), 5-fluorouracil, oxaliplatin plus irinotecan (FOLFOXIRI; *n* = 22), and fluoropyrimidine monotherapy (*n* = 4), and others (*n* = 8). FOLFOXIRI was included only in the FOLFOXIRI and was not included in either the oxaliplatin-based regimen or irinotecan-based regimen. These regimens were combined with or without molecular-targeted agents (Table S2).

The patients were classified into five molecular subtypes based on the presence or absence of mutations in the four most frequently mutated genes as follows: (1) *TP53*-mutation (mut) dominant (*n* = 121), characterized by *TP53* mutations with any genotype of the other three genes either mutated or wild type; (2) *GNAS*-mut focused (*n* = 28), characterized by having *GNAS* mutations, *TP53*-wt, and any *SMAD4*/*KRAS* genotype; (3) *KRAS*-mut focused (*n* = 33), characterized by having *KRAS* mutations, *TP53*-wt, *GNAS*-wt, and any *SMAD4* genotype; (4) *SMAD4*-mut only (*n* = 12), characterized by having *SMAD4* mutations and no mutations in the other three genes; and (5) all wild type (*n* = 51), in which all of these four genes are wild type. The distribution of histology across molecular subtypes is shown in Table 3.

**Table 3:** Distribution of histology across molecular subtypes

Histology	*TP53*-mut dominant (*n* = 155)		*GNAS*-mut focused (*n* = 37)		*KRAS*-mut focused (*n* = 40)		*SMAD4*-mut only (*n* = 12)		All wild type (*n* = 70)	
	*n*	%	*n*	%	*n*	%	*n*	%	*n*	%
Ad	102	65.8	11	29.7	20	50.0	1	8.3	29	41.4
MAd	41	26.5	24	64.9	18	45.0	3	25.0	9	12.9
SRC	4	2.6	0	0	1	2.5	2	16.7	10	14.3
GCA	8	5.2	2	5.4	1	2.5	6	50.0	22	31.4

*Ad* adenocarcinoma, *MAd* mucinous adenocarcinoma, *SRC* signet-ring cell adenocarcinoma, *GCA* goblet cell adenocarcinoma

**Fig. 4:**
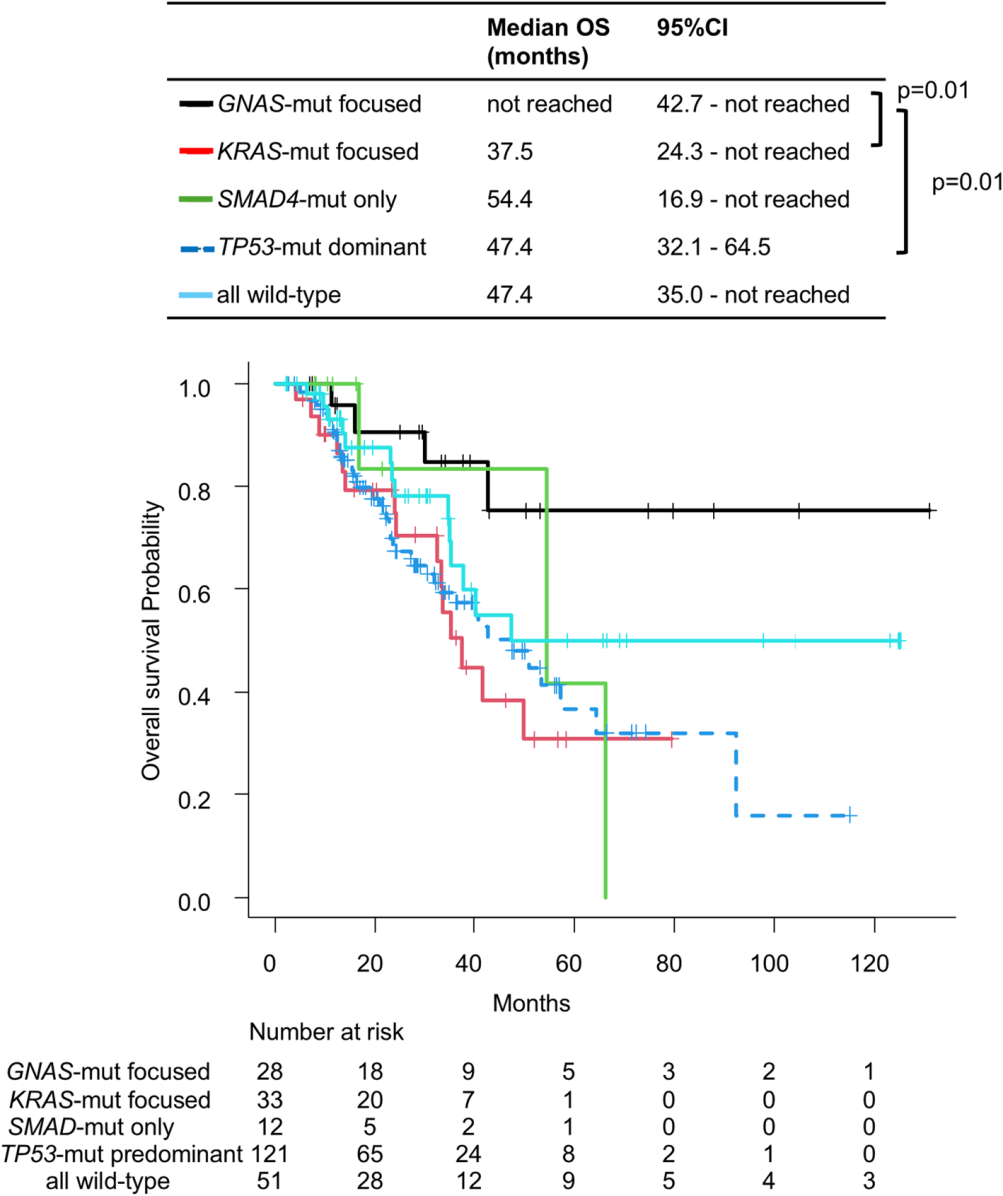
Kaplan–Meier curves for OS according to molecular subtype. The differences were assessed using the log-rank test. *OS* overall survival, *CI* confidence interval

The median follow-up period, calculated using the Kaplan–Meier method, was 32.2 months (95% CI, 28.2–36.7). The median OS of the 245 patients was 49.7 months (95% CI 40.3–66.4). The *p* values for the Wald test were 0.07 for molecular subtype and 0.22 for first-line chemotherapy (Table 4). The Wald test *p* value for first-line chemotherapy was not significant; however, there is a statistical limitation in the analysis of first-line chemotherapy due to the small number of patients in some groups. In the Cox proportional hazards univariable analysis, the patients with *TP53*-mut dominant tumors (median OS, 47.4 months) and *KRAS*-mut focused tumors (37.5 months) showed a worse OS than those with *GNAS*-mut focused tumors (not reached; *p* = 0.01 and *p* = 0.01, respectively) (Fig. 4). The patients who received FOLFOXIRI for first-line chemotherapy may tend to have slightly worse OS than those who received the irinotecan-based regimen (median: 32.8 months vs. 49.7 months). There were no significant differences in OS by histology (Table 4; Fig. S1).

**Table 4:** Cox regression analysis for OS

Characteristics	*n*	Univariable			Multivariable		
		Wald *p*^b^	HR (95% CI)	*p*^c^	Wald *p*^b^	HR (95% CI)	*p*^c^
Age							
Age < 65	168	0.71	1	0.71			
Age ≥ 65	77		1.09 (0.69–1.74)				
Gender							
Male	115	0.81	1	0.81			
Female	130		1.05 (0.68–1.63)				
Histology							
Ad	117	0.15	1				
MAd	83		0.67 (0.41–1.09)	0.11			
SRC	14		1.62 (0.69–3.83)	0.27			
GCA	31		0.67 (0.32–1.43)	0.30			
Molecular subtype							
*GNAS*-mut focused	28	0.07	1		0.05	1	
*TP53*-mut dominant	121		3.63 (1.31–10.11)	0.01		3.65 (1.29–10.34)	0.01
*SMAD4*-mut only	12		2.94 (0.66–13.19)	0.16		2.42 (0.52–11.20)	0.26
*KRAS*-mut focused	33		4.07 (1.35–12.30)	0.01		3.84 (1.24–11.86)	0.02
All wild type	51		2.22 (0.73–6.76)	0.16		1.98 (0.64–6.10)	0.24
TMB							
Low	226	0.15	1	0.15			
High	19		1.63 (0.84–3.16)				
First-line chemotherapy							
Irinotecan-based regimen	45	0.22	1		0.22	1	
Oxaliplatin-based regimen	117		1.11 (0.94–1.64)	0.71		1.42 (0.80–2.52)	0.23
FOLFOXIRI	22		2.08 (1.02–4.23)	0.04		2.35 (1.14–4.88)	0.02
Fluoropyrimidine monotherapy	4		0.58 (0.08–4.38)	0.60		0.93 (0.12–7.13)	0.95
Other regimens	8		1.57 (0.46–5.35)	0.47		2.08 (0.61–7.16)	0.24
Others^a^	49		0.78 (0.36–1.68)	0.53		1.00 (0.46–2.20)	0.99

*OS* overall survival, *HR* hazard ratio, *CI* confidence interval, *Ad* adenocarcinoma, *MAd* mucinous adenocarcinoma, *SRC* signet-ring cell adenocarcinoma, *GCA* goblet cell adenocarcinoma, *TMB* tumor mutation burden

^a^This group includes 45 patients whose chemotherapy regimen was unknown or not administered, and 4 patients whose chemotherapy was discontinued after only one cycle for reasons other than disease progression

^b^Wald test

^c^Cox regression analysis

We included molecular subtype, with a Wald test *p* = 0.07 in the univariable analysis, and first-line chemotherapy, which was clinically inferred to be potentially prognostically relevant, in the multivariable analysis (Table 4). In the Cox proportional hazards multivariable analysis, patients with *TP53*-mut dominant tumors and *KRAS*-mut focused tumors showed a worse OS than those with *GNAS*-mut focused tumors (*p* = 0.01 and *p* = 0.02, respectively).

### TTF

We next examined factors that were associated with chemotherapy efficacy. For this purpose, we used TTF as an index for chemotherapy efficacy. Patients with available TTF data for first-line chemotherapy were included in the analysis.

If one of the combination drugs was suspended for reasons other than disease progression or only fluoropyrimidine drugs were switched to another fluoropyrimidine drug, the treatment was continued, and the TTF was calculated. The TTF data for first-line chemotherapy for unresectable AC were available for 79 patients treated with the oxaliplatin-based regimen, 29 patients treated with the irinotecan-based regimen, 12 patients treated with FOLFOXIRI, and 4 patients treated with the fluoropyrimidine monotherapy; each patient was treated with or without molecular-targeted agents. Table S3 shows the TTF and OS for each regimen.

A Cox proportional hazard analysis for the predictors of TTF and OS for the oxaliplatin-based regimen was conducted (Table S4 and Table 5). TMB was not included as covariates due to low statistical reliability resulting from groups with a small number of patients. Owing to the small number of cases of each molecular subtype, the differences in the TTF and OS were analyzed based on the presence or absence of mutations in one gene rather than the molecular subtypes.

**Table 5:** Univariable analyses of OS for the oxaliplatin-based regimen

Characteristics	*n*	Wald test *p*^a^	Univariable	
			HR (95% CI)	*p*^b^
Clinical valuable				
Age				
Age < 65	27	0.61	1	0.61
Age ≥ 65	52		0.81 (0.37–1.80)	
Gender				
Male	41	0.22	1	0.22
Female	38		0.63 (0.30–1.33)	
Histology				
Ad	33	0.27	1	
MAd	34		0.72 (0.32–1.65)	0.44
SRC or GCA	12		1.68 (0.63–4.48)	0.30
Genotype				
*KRAS*				
WT	39	0.81	1	0.81
Mt	40		1.10 (0.53–2.28)	
*TP53*				
WT	43	0.006	1	0.006
Mt	36		2.93 (1.35–6.33)	
*GNAS*				
WT	64	0.31	1	0.31
Mt	15		0.58 (0.20–1.67)	
*SMAD4*				
WT	60	0.16	1	0.16
Mt	19		1.82 (0.79–4.18)	

*OS* overall survival, *HR* hazard ratio, *CI* confidence interval, *Ad* adenocarcinoma, *MAd* mucinous adenocarcinoma, *SRC* signet-ring cell adenocarcinoma, *GCA* goblet cell adenocarcinoma

^a^Wald test

^b^Cox regression analysis

**Fig. 5:**
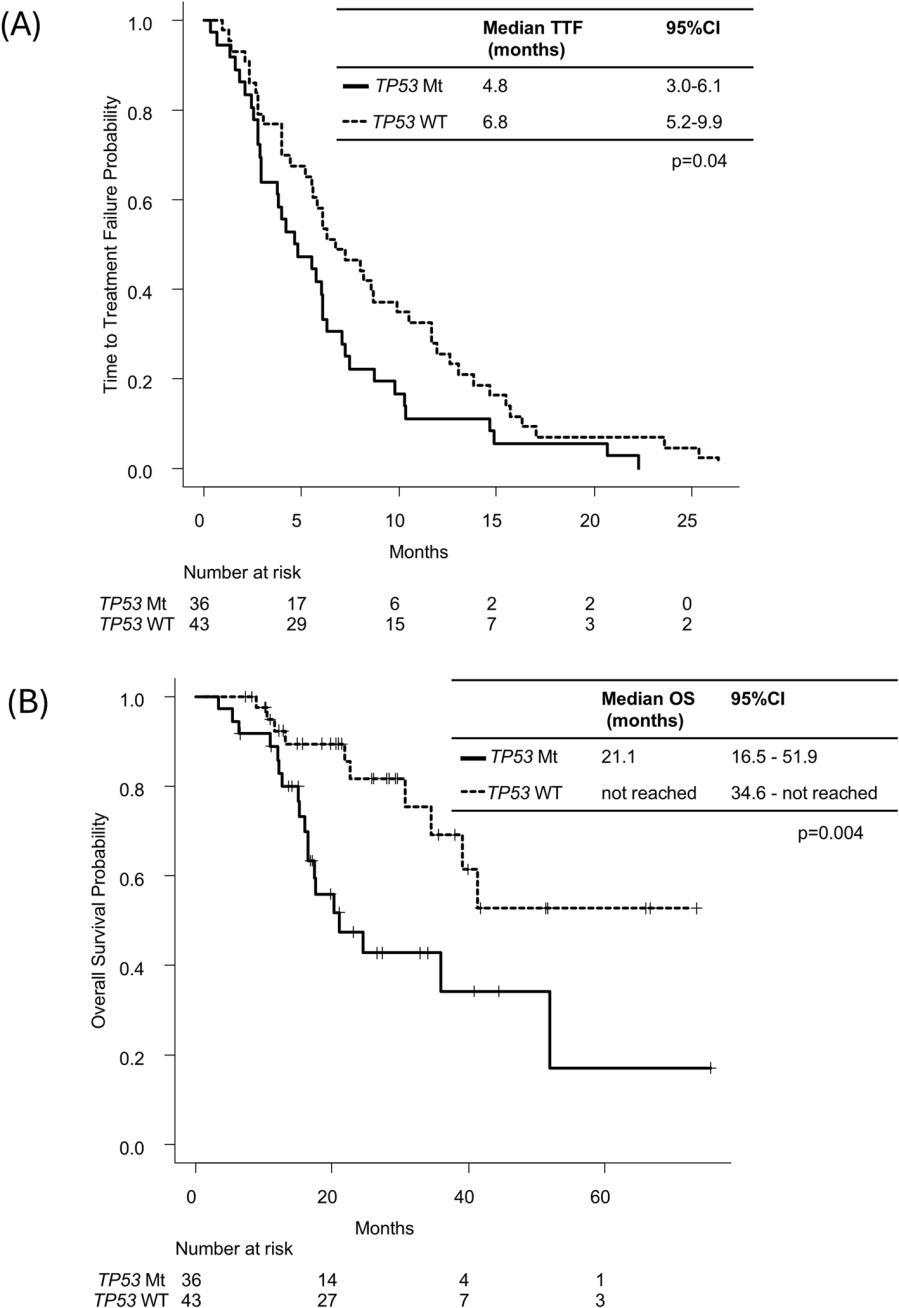
Kaplan–Meier curves for TTF (**a**) and OS (**b**) according to *TP53* mutation status. The differences were assessed using the log-rank test. *TTF* time to treatment failure, *OS* overall survival, *CI* confidence interval

It was revealed that the presence of *TP53* mutation was the only significant prognostic factor in the univariable analysis (Table S4). The patients with *TP53*-mut tumors showed a shorter TTF for the oxaliplatin-based regimen than those with *TP53*-wt tumors (4.8 months vs. 6.8 months; *p* = 0.04) (Fig. 5a).

Regarding the OS for the oxaliplatin-based regimen, *TP53* mutation was considered a significant prognostic factor. The patients with *TP53*-mut tumors showed a worse OS than those with *TP53*-wt tumors (21.1 months vs. not reached; *p* = 0.006 in the univariable analysis) (Fig. 5b; Table 5). Due to the small number of patients, the analysis of the predictors for TTF and OS was not conducted for the irinotecan-based regimen, FOLFOXIRI, or fluoropyrimidine monotherapy.

## Discussion

In the present study, the clinical and genomic characteristics of AC were evaluated, and the impact of the gene mutational patterns on the clinical outcomes of AC was retrospectively analyzed using the data from the Japanese nationwide CGP database, C-CAT database. We found that the top four most commonly detected genetic alterations in AC were mutations in *KRAS*, *TP53*, *SMAD4*, and *GNAS*, indicating different frequencies due to histological types. The molecular subtypes based on gene mutations and first-line chemotherapy were associated with OS. We newly found that *TP53* mutation was associated with poor TTF and OS for the oxaliplatin-based regimen as first-line chemotherapy. Our findings revealed that genetic mutations were associated with the prognosis and chemotherapy efficacy in AC.

Recent studies have suggested that AC is molecularly distinct from CRC [8–12]. The mutation rates for *TP53*, *GNAS*, and *APC* in AC in our study were 49.4%, 17.2%, and 15.3%, respectively. The *TP53* and *APC* mutation rates were lower, while the *GNAS* mutation rate was higher compared with CRC, which is consistent with the findings of previous reports [8, 9].

The prevalence of *KRAS*, *GNAS*, and *TP53* mutations varied by histology. In this study, the trends in the prevalence of *KRAS* and *GNAS* mutations by histological type in AC were generally similar to those in previous reports [8, 11]. However, the *GNAS* mutations in MAd were 33.7% in this study, which was lower than in previous studies (approximately 50%). The *TP53* mutations of this study were mostly prevalent in Ad, followed by MAd, with lower rates in SRC and GCA. Although some differences in the histological classification were observed, the *TP53* mutation patterns were consistent with those of previous reports, wherein the *TP53* mutation rate was highest in Ad and relatively low in MAd and GCA [8, 11].

Previous reports have suggested that the prevalence of gene mutations varied with histologic grade, indicating higher *TP53* mutation rates and lower *GNAS* mutation rates in high-grade AC than in low-grade AC [8, 9]. Information on the histologic grade of AC in this study was not available. It was reported that high-grade AC has *TP53* mutation rates of 28–56% and *GNAS* mutation rates of 10–18%, whereas low-grade AC has *TP53* mutation rates of 7–8% and *GNAS* mutation rates of 49–72% [8, 9]. The *TP53* and *GNAS* mutation rates in this study were similar to those of high-grade tumors, revealing that high-grade tumors may be more prevalent in the present study.

The association between genetic mutations and prognosis in AC has been reported in previous studies. Ang et al. showed that *GNAS* mutations were associated with a better OS, whereas *TP53* mutations were associated with a worse OS. However, the presence of *KRAS* mutations was not significantly associated with survival [8]. Raghav et al. reported that in a univariate analysis, *TP53* and *GNAS* mutations were associated with worse and better survival, respectively [9]. Foote et al. defined distinct molecular lineages from concurrent *GNAS*, *RAS*, and *TP53* mutations and reported that *RAS*-mut predominant tumors had a better OS compared with *TP53*-mut predominant tumors and *GNAS*-mut predominant tumors. *TP53*-mut predominant tumors were associated with a poor prognosis [11]. In the present study, the patients with *TP53*-mut predominant tumors and *KRAS*-mut predominant tumors showed a poorer OS compared with those with *GNAS*-mut predominant tumors. These findings seem to be generally consistent in showing that *TP53* mutations are linked to a poor OS, while *GNAS* mutations are linked to an improved OS. However, the prognostic impact of *KRAS* mutations needs to be further investigated.

However, only a few studies have focused on genetic mutations and response to specific chemotherapies in AC. Ang et al. have reported that the use of irinotecan was associated with a survival advantage in the *KRAS* wild-type AC [8]. Foote et al. have reported that only 1 of 19 patients with *GNAS*-mut predominant AC and 3 of 6 patients with *RAS*-mut predominant AC responded to first-line chemotherapy, suggesting that the latter is more sensitive to chemotherapy [11]. In a study by Pietrantonio et al., the progression-free survival of chemotherapy was shorter in patients with *GNAS*-mut AC than in patients with *GNAS*-wt AC [17]. Our results revealed that *TP53* mutation was associated with poor TTF and OS with the oxaliplatin-based regimen as the first-line chemotherapy, indicating that *TP53* mutation is related to resistance to this regimen.

The present study revealed that the presence of *TP53* mutation was associated with a poor prognosis, particularly in patients who underwent oxaliplatin-based chemotherapy. Recognizing the potential for early resistance and closely monitoring disease progression help patients with *TP53*-mutated AC avoid missing opportunities for treatment changes. By determining the possibility of a poor prognosis, advanced care planning can be implemented. This may enable the patients to mentally prepare themselves and receive the necessary support earlier.

Large and prospective clinical trials are difficult to conduct for AC because of its rarity and its wide variety of histological types [2, 4]. Previous studies on chemotherapy for AC have assessed varying proportions of histologic types using limited sample sizes. The NCCN guidelines recommend treating unresectable advanced AC similarly to unresectable CRC [6, 18, 19]. However, the roles of the anti-VEGF and anti-EGFR antibodies in relation to different chemotherapy regimens remain unclear [7, 20, 21]. Although this study has the bias of patients undergoing CGP tests, this study provides a good overview of the current chemotherapy practices for AC in daily clinical settings in Japan. Despite the small number of patients, this study showed that first-line regimen did not significantly associate with OS. The TRIBE trial revealed that FOLFOXIRI plus bevacizumab was more effective than FOLFIRI plus bevacizumab in right-sided colon cancer [22]. Conversely, data on the use of FOLFOXIRI for AC treatment has been limited. Although the appendix is located on the right side of the colon, it has a different molecular profile from right-sided colon cancer and might respond differently to FOLFOXIRI. Further research is needed to address this.

However, this study has several limitations. First, although the study was based on a nationwide database, the rarity of the diseases limited the number of cases across different histologies, chemotherapy regimens, and molecular subtypes. Since the number of patients would be smaller if the regimens were divided by the presence or absence of anti-VEGF or anti-EGFR antibody combinations, we did not distinguish between the presence or absence of these molecular-targeted agents. Second, due to the nature of the database, which was based on the data manually entered by each attending physician, some data were missing. Third, the pathological diagnosis of the histological type was performed in individual institutions and was not verified by a central review. Fourth, there are histological limitations due to some differences between the WHO classification and the OncoTree classification. Despite these limitations, we believe that our results provide novel insights into the impact of genetic mutations in this rare disease, particularly in relation to chemotherapy sensitivity.

AC and CRC are molecularly different entities, and specialized research efforts in AC are needed. The accumulation of data on AC with sufficient clinical information is necessary. Moreover, analyzing the treatment response by dividing it into different molecular subtypes with different natures may confirm the effectiveness in specific subgroups and help guide the treatment choices. Identification of new therapeutic molecular targets and development of the targeted drug for patients with AC would be desired in future.

In conclusion, our study confirmed the mutation spectrum of AC using data from a national database and revealed that specific genetic mutations were associated with the prognosis and chemotherapeutic response in AC. Considering the limitations of this study, further research is needed to develop better treatment strategies for AC according to molecular markers.

## Supplementary Information

Below is the link to the electronic supplementary material.

Supplementary file1 (PDF 315 KB)

## Data Availability

The data that support the findings of our study are available from the corresponding author upon reasonable request.

## References

[1] Asare EA, Compton CC, Hanna NN et al (2016) The impact of stage, grade, and mucinous histology on the efficacy of systemic chemotherapy in adenocarcinomas of the appendix: analysis of the national cancer data base. Cancer 122(2):213–221. 10.1002/cncr.29744 26506400 10.1002/cncr.29744 PMC4860278

[2] Shaib WL, Assi R, Shamseddine A et al (2017) Appendiceal mucinous neoplasms: diagnosis and management. Oncologist 22(9):1107–1116. 10.1634/theoncologist.2017-0081 28663356 10.1634/theoncologist.2017-0081 PMC5599200

[3] Board WCoTE (2019) WHO classification of tumours digestive system tumours, 5th edn. IARC Press, Lyon

[4] Kelly KJ (2015) Management of appendix cancer. Clin Colon Rectal Surg 28(4):247–255. 10.1055/s-0035-1564433 26648795 10.1055/s-0035-1564433 PMC4655112

[5] Kundra R, Zhang H, Sheridan R et al (2021) OncoTree: a cancer classification system for precision oncology. JCO Clin Cancer Inform 5:221–230. 10.1200/cci.20.00108 33625877 10.1200/CCI.20.00108 PMC8240791

[6] National Comprehensive Cancer Network Colon Cancer (Version 5.2024). https://www.nccn.org/professionals/physician_gls/pdf/colon.pdf. Accessed 9 Sep 2024

[7] Tejani MA, ter Veer A, Milne D et al (2014) Systemic therapy for advanced appendiceal adenocarcinoma: an analysis from the NCCN oncology outcomes database for colorectal cancer. J Natl Compr Cancer Netw 12(8):1123–1130. 10.6004/jnccn.2014.0109 10.6004/jnccn.2014.0109 25099444

[8] Ang CS, Shen JP, Hardy-Abeloos CJ et al (2018) Genomic landscape of appendiceal neoplasms. JCO Precis Oncol. 10.1200/po.17.00302 32913983 10.1200/PO.17.00302 PMC7446344

[9] Raghav K, Shen JP, Jácome AA et al (2020) Integrated clinico-molecular profiling of appendiceal adenocarcinoma reveals a unique grade-driven entity distinct from colorectal cancer. Br J Cancer 123(8):1262–1270. 10.1038/s41416-020-1015-3 32733093 10.1038/s41416-020-1015-3 PMC7553941

[10] Tokunaga R, Xiu J, Johnston C et al (2019) Molecular profiling of appendiceal adenocarcinoma and comparison with right-sided and left-sided colorectal cancer. Clin Cancer Res 25(10):3096–3103. 10.1158/1078-0432.Ccr-18-3388 30692096 10.1158/1078-0432.CCR-18-3388 PMC6886223

[11] Foote MB, Walch H, Chatila W et al (2023) Molecular classification of appendiceal adenocarcinoma. J Clin Oncol 41(8):1553–1564. 10.1200/jco.22.01392 36493333 10.1200/JCO.22.01392 PMC10043565

[12] Wald AI, Pingpank JF, Ongchin M et al (2023) Targeted next-generation sequencing improves the prognostication of patients with disseminated appendiceal mucinous neoplasms (*Pseudomyxoma Peritonei*). Ann Surg Oncol 30(12):7517–7526. 10.1245/s10434-023-13721-y 37314541 10.1245/s10434-023-13721-y

[13] Valasek MA, Thung I, Gollapalle E et al (2017) Overinterpretation is common in pathological diagnosis of appendix cancer during patient referral for oncologic care. PLoS One 12(6):e0179216. 10.1371/journal.pone.0179216 28591173 10.1371/journal.pone.0179216 PMC5462425

[14] Kohno T, Kato M, Kohsaka S et al (2022) C-CAT: the national datacenter for cancer genomic medicine in Japan. Cancer Discov 12(11):2509–2515. 10.1158/2159-8290.Cd-22-0417 36321305 10.1158/2159-8290.CD-22-0417 PMC9762342

[15] Mukai Y, Ueno H (2021) Establishment and implementation of cancer genomic medicine in Japan. Cancer Sci 112(3):970–977. 10.1111/cas.14754 33289217 10.1111/cas.14754 PMC7935799

[16] Gao J, Aksoy BA, Dogrusoz U et al (2013) Integrative analysis of complex cancer genomics and clinical profiles using the cBioPortal. Sci Signal 6(269):1. 10.1126/scisignal.2004088 10.1126/scisignal.2004088 PMC4160307 23550210

[17] Pietrantonio F, Berenato R, Maggi C et al (2016) GNAS mutations as prognostic biomarker in patients with relapsed peritoneal pseudomyxoma receiving metronomic capecitabine and bevacizumab: a clinical and translational study. J Transl Med 14(1):125. 10.1186/s12967-016-0877-x 27154293 10.1186/s12967-016-0877-x PMC4859944

[18] Hiraide S, Komine K, Sato Y et al (2020) Efficacy of modified FOLFOX6 chemotherapy for patients with unresectable pseudomyxoma peritonei. Int J Clin Oncol 25(4):774–781. 10.1007/s10147-019-01592-x 31823151 10.1007/s10147-019-01592-x PMC7118031

[19] Strach MC, Sutherland S, Horvath LG et al (2022) The role of chemotherapy in the treatment of advanced appendiceal cancers: summary of the literature and future directions. Ther Adv Med Oncol 14:17588359221112478. 10.1177/17588359221112478 35898968 10.1177/17588359221112478 PMC9310237

[20] Choe JH, Overman MJ, Fournier KF et al (2015) Improved survival with anti-VEGF therapy in the treatment of unresectable appendiceal epithelial neoplasms. Ann Surg Oncol 22(8):2578–2584. 10.1245/s10434-014-4335-9 25582740 10.1245/s10434-014-4335-9

[21] Shapiro JF, Chase JL, Wolff RA et al (2010) Modern systemic chemotherapy in surgically unresectable neoplasms of appendiceal origin: a single-institution experience. Cancer 116(2):316–322. 10.1002/cncr.24715 19904805 10.1002/cncr.24715

[22] Cremolini C, Antoniotti C, Lonardi S et al (2018) Primary tumor sidedness and benefit from FOLFOXIRI plus bevacizumab as initial therapy for metastatic colorectal cancer. Retrospective analysis of the TRIBE trial by GONO. Ann Oncol 29(7):1528–1534. 10.1093/annonc/mdy140 29873679 10.1093/annonc/mdy140

