# Supplementary material for: Impact of genetic mutations on prognosis and chemotherapy efficacy in advanced appendiceal carcinoma: insights from the nationwide Japanese comprehensive genomic profiling test database
Source: Int J Clin Oncol. 2025 Feb 28;30(5):914–25. doi: 10.1007/s10147-025-02724-2 (PMC12014699; doi:10.1007/s10147-025-02724-2)
Supplement: Supplementary file 1 — Supplementary file1 (PDF 315 KB) [file 10147_2025_2724_MOESM1_ESM.pdf]

Supplementary materials

Title: Impact of genetic mutations on prognosis and chemotherapy efficacy in advanced appendiceal carcinoma : insights from the nationwide Japanese comprehensive genomic profiling test database

Journal name: International Journal of Clinical Oncology

Authors: Sakura Hiraide Taniguchi, Masanobu Takahashi, Shih-Wei Chiu, Keigo Komine, Ryunosuke Numakura, Yuya Yoshida, Yuki Kasahara, Kota Ouchi, Hiroo Imai, Ken Saijo, Hidekazu Shirota, Chikashi Ishioka

Correspondence: Masanobu Takahashi

Department of Clinical Oncology, Tohoku University Graduate School of Medicine

4-1, Seiryo-machi, Aoba-ku, Sendai, Miyagi 980-8575, Japan

E-mail: masanobu.takahashi.a7@tohoku.ac.jp

Table S1 Frequency of gene mutations in our patients

Gene	All patients (n=314)		Patients with OS data (n=245)	
	n	%	n	%
KRAS	165	52.5	126	51.4
TP53	155	49.4	121	49.4
SMAD4	59	18.8	42	17.1
GNAS	54	17.2	41	16.7
APC	48	15.3	34	13.9
PIK3CA	41	13.1	33	13.5
STK11	25	8.0	19	7.8
MYC	23	7.3	15	6.1
SOX9	20	6.4	16	6.5
ARID1A	19	6.1	16	6.5
BRAF	19	6.1	15	6.1
TGFBR2	16	5.1	15	6.1
RNF43	13	4.1	10	4.1
FBXW7	13	4.1	12	4.9
ERBB2	12	3.8	10	4.1
CDK8	12	3.8	11	4.5
ATM	12	3.8	11	4.5
AMER1	10	3.2	6	2.4
FLT3	10	3.2	8	3.3
ASXL1	10	3.2	7	2.9
NRAS	9	2.9	8	3.3
CARD11	8	2.5	7	2.9
MSH6	8	2.5	5	2.0
CDKN2A	8	2.5	8	3.3
KDM6A	8	2.5	5	2.0
SMO	8	2.5	7	2.9
RICTOR	8	2.5	7	2.9
PTEN	7	2.2	3	1.2
ERBB3	7	2.2	3	1.2
BCOR	7	2.2	5	2.0
PBRM1	7	2.2	6	2.4
NF1	7	2.2	5	2.0
AKT1	6	1.9	5	2.0
KDM5A	6	1.9	5	2.0
BRCA2	6	1.9	4	1.6

KMT2D	6	1.9	6	2.4
TET2	6	1.9	6	2.4
CCND2	6	1.9	4	1.6
AURKA	5	1.6	4	1.6
MED12	5	1.6	3	1.2
CCND1	5	1.6	4	1.6
PTPN11	5	1.6	3	1.2
DNMT3A	5	1.6	3	1.2
AKT2	5	1.6	4	1.6
BRCA1	4	1.3	3	1.2
MAP2K1	4	1.3	2	0.8
CDKN2B	4	1.3	4	1.6
RAD51B	4	1.3	3	1.2
FGFR1	4	1.3	3	1.2
CCNE1	4	1.3	2	0.8
PALB2	4	1.3	4	1.6
SMAD2	4	1.3	2	0.8
FGF19	4	1.3	3	1.2
MUTYH	4	1.3	2	0.8
BCL2L1	3	1.0	2	0.8
SRC	3	1.0	2	0.8
CTCF	3	1.0	2	0.8
CCND3	3	1.0	3	1.2
JAK2	3	1.0	3	1.2
ERCC4	3	1.0	3	1.2
MTOR	3	1.0	3	1.2
ERBB4	3	1.0	2	0.8
BCORL1	3	1.0	3	1.2
ATR	3	1.0	3	1.2
NOTCH1	3	1.0	2	0.8
NOTCH3	3	1.0	2	0.8
CHEK2	3	1.0	3	1.2
CDK4	3	1.0	2	0.8
AXL	3	1.0	2	0.8
AKT3	3	1.0	3	1.2
SPEN	3	1.0	2	0.8
MAPK1	3	1.0	3	1.2
FGFR2	3	1.0	2	0.8
CDH1	3	1.0	2	0.8

BRIP1	3	1.0	1	0.4
TSC2	2	0.6	2	0.8
EPHA3	2	0.6	1	0.4
FGF6	2	0.6	0	0.0
VEGFA	2	0.6	2	0.8
EGFR	2	0.6	2	0.8
PPP2R1A	2	0.6	1	0.4
INPP4B	2	0.6	2	0.8
AR	2	0.6	2	0.8
MITF	2	0.6	2	0.8
SF3B1	2	0.6	1	0.4
BARD1	2	0.6	2	0.8
TBX3	2	0.6	2	0.8
MDM2	2	0.6	0	0
CREBBP	2	0.6	2	0.8
EP300	2	0.6	2	0.8
RB1	2	0.6	2	0.8
SETD2	2	0.6	2	0.8
KMT2A	2	0.6	1	0.4
MLH1	2	0.6	2	0.8
NOTCH2	2	0.6	1	0.4
WT1	2	0.6	1	0.4
IDH1	2	0.6	2	0.8
CTNNB1	2	0.6	2	0.8
CDKN1B	2	0.6	1	0.4
PIK3R1	2	0.6	2	0.8
RAD51D	2	0.6	2	0.8
HGF	1	0.3	1	0.4
MSH3	1	0.3	1	0.4
PTCH1	1	0.3	1	0.4
MET	1	0.3	1	0.4
JAK3	1	0.3	1	0.4
MEF2B	1	0.3	1	0.4
MAP2K2	1	0.3	0	0
ALK	1	0.3	1	0.4
MYCN	1	0.3	1	0.4
NF2	1	0.3	1	0.4
ALK-SPTI	1	0.3	1	0.4
SPTBN1-	1	0.3	1	0.4

TSC1	1	0.3	1	0.4
MAP2K4	1	0.3	0	0.0
STAT3	1	0.3	1	0.4
CDK6	1	0.3	1	0.4
CSF3R	1	0.3	1	0.4
DIS3	1	0.3	1	0.4
GATA3	1	0.3	1	0.4
HNF1A	1	0.3	1	0.4
SUFU	1	0.3	1	0.4
ARID2	1	0.3	1	0.4
RARA-PS[illegible]	1	0.3	1	0.4
TEK	1	0.3	1	0.4
DDR2	1	0.3	1	0.4
IKBKE	1	0.3	1	0.4
MCL1	1	0.3	1	0.4
MDM4	1	0.3	1	0.4
NTRK1	1	0.3	1	0.4
ERRFI1	1	0.3	1	0.4
PARP1	1	0.3	1	0.4
ATRX	1	0.3	1	0.4
CIC	1	0.3	1	0.4
EMSY	1	0.3	1	0.4
JAK1	1	0.3	1	0.4
EZH2	1	0.3	1	0.4
KDR	1	0.3	1	0.4
RAC1	1	0.3	1	0.4
PMS2	1	0.3	1	0.4
TNFAIP3	1	0.3	1	0.4
MAP3K1	1	0.3	1	0.4
MRE11	1	0.3	1	0.4
RAD51C	1	0.3	1	0.4
CBL	1	0.3	1	0.4
MPL	1	0.3	1	0.4
ETV6-LO[illegible]	1	0.3	0	0
SPOP	1	0.3	0	0
CDK12	1	0.3	1	0.4
FLT1	1	0.3	0	0
SMARCA[illegible]	1	0.3	1	0.4
GOPC-R[illegible]	1	0.3	1	0.4

PAX5	1	0.3	0	0
INPPL1	1	0.3	0	0
PTPRT	1	0.3	0	0
FANCL	1	0.3	0	0
BCL2	1	0.3	0	0

OS overall survival

Table S2 First-line chemotherapy regimens

Combined molecular targeted drugs	Oxaliplatin-based regimen (n=117)	Irinotecan-based regimen (n=45)	FOLFOXIRI (n=22)	Fluoropyrimidime monotherapy (n=4)
anti-VEGF antibodies	86	38	19	0
anti-EGFR antibodies	5	4	1	2
No	26	3	2	2

Table S3 TTF and OS of primary chemotherapy for unresectable appendiceal carcinoma

	Oxaliplatin-based regimen (n=79)	Irinotecan-based regimen (n=29)	FOLFOXIRI (n=12)	Fluoropyrimidime monotherapy (n=4)
Median TTF (months)	6.0	5.6	9.4	8.2
Median OS (months)	39.1	40	32.4	not applicable

TTF time to treatment failure, *OS* overall survival

Table S4 Univariable analyses of TTF for oxaliplatin-based regimen

			n	Wald	Univariable	
				p^{a}	HR (95%CI)	p^{b}
Clinical variable	Gender	Male	41	0.11	1	0.11
		Female	38		0.69 (0.44-1.08)	
	Age	Age < 65	27	0.88	1	0.88
		Age ≥ 65	52		0.97 (0.60-1.54)	
	Histology	Ad	33	0.5	1	
		MAd	34		0.90 (0.56-1.46)	0.67
		SRC or GCA	12		0.66 (0.33-1.32)	0.24
Genotype	*KRAS*	WT	39	0.8	1	0.8
		Mt	40		1.06 (0.68-1.66)	
	TP53	WT	43	0.04	1	0.04
		Mt	36		1.60 (1.01-2.52)	
	GNAS	WT	64	0.68	1	0.68
		Mt	15		0.89 (0.50-1.57)	
	SMAD4	WT	60	0.75	1	0.75
		MT	19		1.09 (0.65-1.83)	

TTF time to treatment failure, *HR* hazard ratio, *CI* confidence interval

Ad adenocarcinoma, *MAd* mucinous adenocarcinoma, *SRC* signet-ring cell adenocarcinoma, *GCA* goblet cell adenocarcinoma

[a] Wald test.

[b] Cox regression analysis.

Fig. S1 Kaplan-Meier curves for OS according to histological subtype

	Median OS (months)	95%CI
Ad	49.7	32.8 – 66.4
MAd	54.4	41.6 - not reached
SRC	33.3	16.9 - not reached
GCA	not reached	35.3 - not reached

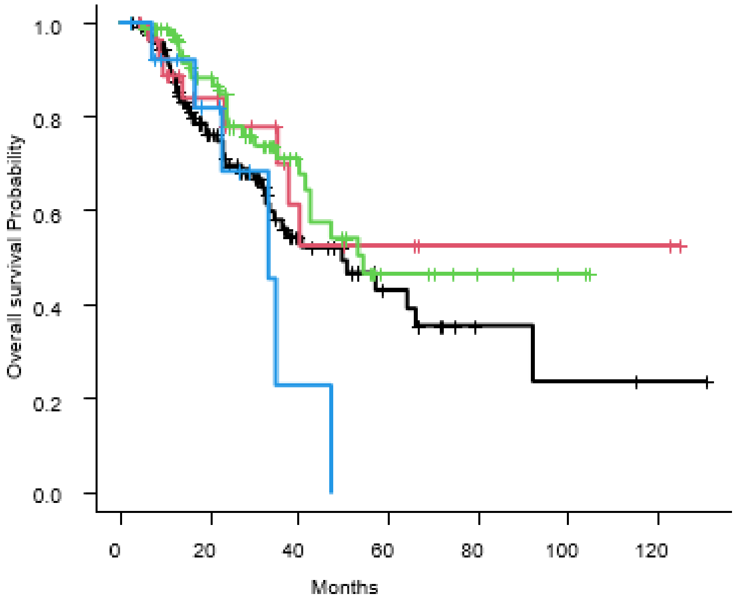

Number at risk

Ad	117	59	25	11	3	2	1
MAd	83	55	21	8	4	2	0
SRC	14	7	1	0	0	0	0
GCA	31	15	7	5	3	3	3

Fig. S1 Kaplan–Meier curves for OS according to histological subtype. The differences were assessed using the log-rank test. *OS* overall survival, *CI* confidence interval, *Ad* adenocarcinoma, *MAd* mucinous adenocarcinoma, *SRC* signet-ring cell adenocarcinoma, *GCA* goblet cell adenocarcinoma.
